# Teenage Pregnancy and Its Associated Factors Among Girls Aged 13–19 Years in Apac District, Uganda: A Community‐Based Cross‐Sectional Study

**DOI:** 10.1002/hsr2.70471

**Published:** 2025-02-16

**Authors:** Ewach Gracious Faith, Emmanuel Asher Ikwara, Musinguzi Marvin, Isaac Isiko

**Affiliations:** ^1^ Department of Environmental Health and Disease Control, Faculty of Public Health Lira University Lira Uganda; ^2^ Department of Public Health and Nutrition, Faculty of Health Sciences Victoria University Kampala Uganda; ^3^ Child Health & Development Centre Department, School of Medicine, College of Health Sciences Makerere University Kampala Uganda; ^4^ Department of Community Medicine, Axel Pries Institute of Public Health and Biomedical Sciences Nims University Jaipur Rajasthan India

**Keywords:** cultural factors, early marriage, sociodemographics, teenage pregnancy, Uganda

## Abstract

**Background:**

This study examined teenage pregnancy among girls aged 13–19 years in Apac District, Uganda, focusing on socioeconomic, cultural, and behavioral factors. It aimed to identify prevalence and key contributors to inform targeted interventions and improve adolescent reproductive health.

**Methods:**

This study employed a descriptive cross‐sectional design in Apac District, Uganda. It surveyed 432 teenage girls (13–19) using structured questionnaires to examine sociodemographic, behavioral, and sociocultural factors affecting teenage pregnancy. Data analysis was conducted in Excel and SPSS.

**Results:**

This study identified critical factors influencing teenage pregnancy among girls aged 13–19. A striking 84.1% of pregnant adolescents are aged 13–14, demonstrating age as a primary risk factor. Educational disparities emerge, with no formal education significantly lowering pregnancy odds (AOR: 0.16, CI: 0.03–0.80). Conversely, higher parental education correlates with increased risk (AOR: 3.50, CI: 1.50–8.15). Cultural influences are notable; Muslim (AOR: 4.60, CI: 1.56–13.58) and Protestant affiliations (AOR: 2.09, CI: 1.08–4.09) elevate risks, while early marriage (AOR: 7.57, CI: 3.44–16.64) and schooling challenges (AOR: 6.97, CI: 3.18–15.31) further exacerbate vulnerability.

**Conclusion:**

This study highlights the critical factors contributing to teenage pregnancy in Apac District, Uganda, including younger age, educational disparities, and cultural affiliations. Early marriage and schooling challenges also increase vulnerability, emphasizing the need for targeted interventions to improve maternal health outcomes.

**Abbreviations**
HIV: human immunodeficiency virus
NGO: nongovernmental organization
SDG: sustainable development goal
STD: sexually transmitted disease
UNICEF: United Nations Children's Fund

## 1 Introduction

Teenage pregnancy remains a significant public health concern worldwide, particularly in sub‐Saharan Africa [1], where it contributes to high maternal morbidity and mortality rates [2]. In Uganda, the incidence of teenage pregnancy is alarmingly high, with 25% of girls aged 15–19 having begun childbearing, and teenage pregnancy being a major determinant of school dropout and poor health outcomes [3, 4]. Sub‐Saharan Africa, in particular, has the highest prevalence of teenage pregnancies globally [5]. In 2013, teenage mothers accounted for over half of all births in the region, with certain countries experiencing up to 101 births/1000 women aged 15–19—nearly double the global average [6]. Fourteen out of the 15 countries with the highest teenage birth rates (over 30% of women giving birth before the age of 18) are in sub‐Saharan Africa, including Uganda, Niger, and Mozambique [7].

Teenage pregnancy in Uganda is influenced by a combination of socioeconomic, cultural, and educational factors that exacerbate the vulnerability of adolescent girls to early childbearing, especially in rural districts like Apac [8]. Limited access to sexual and reproductive health education, early marriage, poverty, and gender inequality are key contributors to the high rates of teenage pregnancy in these areas [9]. These factors not only increase the risk of unintended pregnancies but also pose significant barriers to the health and well‐being of adolescent girls, limiting their potential for education and future economic opportunities [10].

The health and social consequences of teenage pregnancy are far‐reaching. Teenage pregnancies are associated with higher maternal and child health risks, as pregnancy and childbirth are leading causes of death among girls aged 15–19 globally, particularly in low‐ and middle‐income countries where the adolescent birth rate is much higher than in high‐income countries [11]. Furthermore, teenage pregnancy limits girls' educational attainment, disrupts their access to economic opportunities, and perpetuates cycles of poverty [12]. In regions with limited access to contraceptive methods and family planning services, the risk of rapid population growth is further exacerbated, placing additional strain on health systems and social services.

Research conducted in other regions of Uganda and sub‐Saharan Africa has identified factors such as early sexual debut, peer pressure, and early marriage as significant contributors to teenage pregnancy [13]. In Uganda, studies have shown that adolescent girls who experience early marriage are at a significantly higher risk of early pregnancy, which in turn perpetuates poor health and socioeconomic outcomes [14]. Additionally, studies have highlighted the barriers posed by limited access to sexual and reproductive health education, with rural areas often facing higher rates of teenage pregnancy due to lower levels of educational attainment and fewer resources for family planning [15]. However, these findings do not fully capture the local context in Apac District, where sociocultural dynamics and access to educational resources may differ markedly from those in more urbanized regions.

This study, therefore, aims to fill this gap by exploring the socioeconomic, cultural, and behavioral factors associated with teenage pregnancy among girls aged 13–19 years in Apac District, Uganda. Through a community‐based cross‐sectional study, this research will examine the prevalence of teenage pregnancy and identify key factors contributing to its high rates in the region. The identification of these factors is crucial for designing targeted interventions that can reduce teenage pregnancies and improve the reproductive health of adolescent girls in Apac.

While Uganda has national policies in place to address teenage pregnancy, such as the National Adolescent Sexual and Reproductive Health Policy [16], the lack of region‐specific data has often hindered the effectiveness of these policies. This study seeks to answer the following research questions: What are the socioeconomic, cultural, and behavioral factors associated with teenage pregnancy in Apac District? What is the prevalence of teenage pregnancy among girls aged 13–19 years in this region? The findings of this study will provide essential data to inform localized interventions and strategies to combat teenage pregnancy in rural areas of Uganda.

The significance of this study extends beyond the local context, with the potential to influence both regional and national policies on adolescent reproductive health. By utilizing a community‐based approach, this research will gather primary data that reflect the experiences of adolescent girls in Apac, contributing to the ongoing discourse on adolescent health in Uganda and sub‐Saharan Africa.

## 2 Methods and Tools

### 2.1 Study Site and Settings

The study was conducted in Apac District, located in Northern Uganda, Lango subregion. According to district statistics, Apac district comprises 8 subcounties, 64 parishes, and 341 villages. The National Population and Housing Census of 2014 reported a total population of 368,626 in Apac District, with 180,995 males and 187,631 females. There were 65,633 individuals in the 10–15 age group and 84,106 in the 15–24 age group, which includes the target population of this study.

### 2.2 Study Design and Study Population

This study employed a descriptive cross‐sectional design and utilized quantitative data collection and analysis techniques. The selection of a quantitative approach allowed for the exploration of various variables. The study targeted teenage girls between the ages of 13 and 19 years living in the Apac District at the time of the study 23.

### 2.3 Study Procedure

The research proposal was reviewed and approved by the Lira University Faculty of Public Health after being submitted to the university supervisor. Subsequently, permission was obtained from local council authorities in the study area. Before data collection, informed consent from the guardians/parents of the participants was sought and informed assent from the participants themselves. Researcher‐administered structured questionnaires were used for data collection, and a report of findings has been prepared for future dissemination and utilization.

### 2.4 Eligibility Criteria (Inclusion and Exclusion)

All teenage girls between 13 and 19 were eligible to participate. Those who were seriously ill, or mentally ill at the time of the study, teenage girls aged 18 and above who did not consent to participate, and those aged 13–18 whose parents did not provide consent were excluded.

### 2.5 Sample Size Determination

The study employed the Keish–Leslie formula (1967) to calculate the sample size, ensuring that the selected sample was representative and provided sufficient statistical power for analysis. A minimum of 432 participants was determined based on this calculation. Below is the detailed justification and computation:

The formula used for sample size calculation is:

$$n=\left[\frac{\mathrm{ZPQ}}{d^{2}}\right]\mathrm{def}$$

where:

- *P* = 25% (proportion of the population expected to exhibit the characteristic of interest),
- *Q* = 75% (complementary proportion, *Q* = 1 − *P*),
- *Z* = 1.96 (*Z*‐score corresponding to a 95% confidence level),
- *d* = 0.05 (margin of error, commonly set at 5%).

Substituting these values into the formula:

$$n=1.5\left(1.96^{2}\times 0.25\times 0.75\right)/0.05^{2}432\;\mathrm{participants}$$

Thus, the sample size was calculated to be 432 participants, ensuring sufficient statistical power to detect meaningful effects while maintaining a reasonable margin of error (5%) and confidence (95%).

### 2.6 Data Collection Transparency

Upon review of the data collection process, 450 questionnaires were initially distributed. However, 11 questionnaires were excluded due to incomplete responses. Additionally, 7 questionnaires were randomly excluded to ensure the final sample represented a diverse range of participants and excluded outliers or extreme responses that might distort the results. This resulted in a final sample of 432 valid responses. Nonresponses were tracked, and follow‐up reminders were sent to participants who did not initially respond and a final cut‐off date for data collection was set to finalize the sample size.

#### 2.6.1 Sampling Methods and Techniques

A multistage sampling technique was used in three stages:

1. **Stage 1**: Two subcounties were randomly selected from the eight in the district using simple random sampling (lottery method).
2. **Stage 2**: From each of the selected subcounties, two parishes were randomly chosen using simple random sampling.
3. **Stage 3**: At the parish level, two villages were selected from each parish using systematic sampling.

The population of each selected village was determined by the Local Council Chairpersons (LCIs). The number of participants per village was calculated proportionally, based on the village's population relative to the total population of all selected villages and the overall sample size. Finally, a random sampling method was used to select the study participants from each village.

### 2.7 Data Collection Method and Instrument

The study utilized a researcher‐administered questionnaire to collect quantitative data on sociodemographic, behavioral, and sociocultural factors associated with teenage pregnancy in Apac District. The questionnaire, available in both English and Lango, was developed through a review of relevant literature and studies from nearby districts, ensuring cultural relevance. It was structured into three sections: sociodemographic factors, behavioral factors (such as age of first sexual encounter, contraception use, early marriage, and sexual partners), and sociocultural factors (including the ability to discuss sexual matters with parents). The questionnaire underwent pretesting with a similar demographic, leading to revisions for clarity and cultural sensitivity. Sensitive questions were carefully worded, and participants were assured of confidentiality and the option to withdraw. Researchers consulted local experts to address challenges in phrasing questions appropriately.

### 2.8 Data Cleaning and Data Analysis

Data entry and cleaning were performed using Microsoft Excel. SPSS Statistics 26.0 was used for data analysis. The descriptive characteristics of participants were summarized using frequencies and percentages in Tables 1 and 2 by cross‐tabulations using the *χ* ^2^ test. Significant variables at cross‐tabulation were fed into a multivariate analysis model and Adjusted odds ratios at a significance level of *p* < 0.05% and 95% CI were used to predict teenage pregnancy and recorded in Table 3.

**Table 1:** Sociodemographic characteristics of the study participants.

Sociodemographic variables	Teenage pregnancy *N* = 432		
	Pregnant (*n* = 166) *n*(%)	Not pregnant (*n* = 266) *n*(%)	*p* value
Age			< 0.001
13–14	58 (84.1)	11 (15.9)	
15–17	61 (43)	81 (57)	
18–19	44 (19.9)	177 (80.1)	
Participant level of education			< 0.001
Primary	113 (46.7)	129 (53.3)	
Secondary	44 (26)	125 (74)	
Not at school	15 (71.4)	6 (28.6)	
No education	28 (20.6)	108 (79.4)	
Parents level of education			0.005
Primary	48 (36.1)	85 (63.9)	
Secondary	58 (48.7)	61 (51.3)	
Postsecondary	29 (65.9)	15 (34.1)	
Religion			0.081
Catholic	45 (28.3)	114 (71.7)	
Protestant	78 (37)	133 (63)	
Muslim	29 (64.4)	16 (35.6)	
PAG	11 (64.7)	6 (35.3)	
Residence			
Rural	116 (40)	174 (60)	
Urban	95 (67)	47 (33)	0.198

**Table 2:** Behavioral and sociocultural factors‐related characteristics of study participants.

Behavioral and sociocultural variable	Teenage pregnancy *N* = 432		
	Pregnant (*n* = 166) *n*(%)	Not pregnant (*n* = 266) *n*(%)	*p* value
Peer pressure			0.374
No	88 (35.9)	157 (64.1)	
Yes	75 (40.1)	112 (59.9)	
Contraceptive use			
No	157 (39.9)	236 (60.1)	
Yes	5 (13.2)	33 (86.8)	
Early marriage			< 0.001
No	80 (25.8)	230 (74.2)	
Yes	83 (68)	39 (32)	
Multiple sexual partners			< 0.001
No	86 (26.3)	241 (73.7)	
Yes	77 (73.3)	28 (26.7)	
Challenges with schooling			< 0.001
No	83 (26.9)	225 (73.1)	
Yes	80 (64.5)	44 (35.5)	
Having strict parents			0.112
No	75 (34.1)	145 (65.9)	
Yes	88 (41.5)	124 (58.5)	
School rules			0.817
No	86 (37.2)	145 (62.8)	
Yes	77 (38.3)	124 (61.7)	
My cultural beliefs allow me to discuss sexual matters with my parents			< 0.001
No	113 (45)	138 (55)	
Yes	49 (27.2)	131 (72.8)	
My culture allows sex before the age of 18			0.003
No	157 (39.9)	236 (60.1)	
Yes	5 (13.2)	33 (86.8)	

**Table 3:** Factors associated with teenage pregnancy among girls.

Variable	AOR (CI)	*p* value
Age group		
13–14	Ref	
15–17	0.17 (0.06–0.46)	< 0.001
18–19	0.04 (0.01–0.12)	< 0.001
Education level		
Primary	Ref	
Secondary	1.42 (0.63–3.20)	0.398
None	0.162 (0.03–0.80)	0.025
Parents education level		
None	Ref	
Primary	1.69 (0.76–3.78)	0.202
Secondary	3.50 (1.5–8.15)	0.004
Postsecondary	3.36 (0.92–11.62)	0.067
Religion		
Catholic	Ref	
Protestant	2.09 (1.08–4.09)	0.029
Muslim	4.60 (1.56–13.58)	0.006
PAG	2.49 (0.41–15.16)	0.321
Contraceptive use		
No	Ref	
Yes	0.04 (0.01–0.12)	< 0.001
Early marriage		
No	Ref	
Yes	7.57 (3.44–16.64)	< 0.001
Challenges with schooling		
No	Ref	
Yes	6.97 (3.18–15.31)	< 0.001
Having strict parents		
No	Ref	
Yes	6.37 (2.54–15.98)	< 0.001
Culture allows sex before 18 years of age		
No	Ref	
Yes	0.16 (0.038–0.64)	0.01

### 2.9 Measurement of Study Variables

The dependent variable in this study was teenage pregnancy, measured by responses to the questions “Are you currently pregnant?” and “Have you ever been pregnant?.”

These questions were coded as binary (Yes = 1, No = 0) to create the outcome variable for instance, if a respondent responded “Yes” to any of the questions, the outcome variable shall be coded as 1 (every respondent who has ever had teenage pregnancy or currently pregnant). If the least number of affirmative answers was received from the respondent who answered the question as “No,” then the outcome variable was coded as 0 which denotes never had teenage pregnancy before or not pregnant.

Independent variables were categorized into sociodemographic, behavioral, and sociocultural factors, guided by the Social‐Ecological and Health Belief Models [17].

**Sociodemographic factors**: Included age, participant level of education, parents level of education, religion, and place of residence.

**Behavioral factors and sociocultural factors:** This included peer pressure, contraceptive use, early marriages, multiple sexual partners, challenges with schooling, having strict parents, school rules, my cultural beliefs allow me to discuss sexual matters with my parents, and my culture allows sex before the age of 18.

The selection of these variables was informed by both theoretical relevance and empirical findings, with statistical significance as one of the criteria. The study's framework captured the multilayered influences on teenage pregnancy in Apac District, Uganda, enhancing the depth of analysis.

### 2.10 Quality Control (Validity and Reliability)

A 1‐day training session was conducted with four research assistants. A pretest was conducted with 20% of the respondents using the questionnaires to ensure the tool's validity and suitability in the study area. Questions that were found to be difficult to understand or irrelevant to the study were adjusted or removed. Data quality checks were carried out continuously throughout the study, during data collection, and processing for completeness and consistency. The completeness and consistency of the data tool were also checked.

The questionnaire used for data collection was adapted and modified from reliable sources to be suitable for collecting data from the participants.

## 3 Results

### 3.1 Sociodemographic Factors

Table 1 highlights sociodemographic factors linked to teenage pregnancy. A significant majority (84.1%) of pregnant teenagers are aged 13–14, indicating younger age as a key risk factor. Education levels also played a role, with 71.4% of out‐of‐school teens pregnant, while only 26% of those with secondary education were pregnant. Parental education showed mixed results, with higher rates of pregnancy among teens with postsecondary educated parents. Religious affiliation revealed that 64.4% of pregnant teens were Muslim. Urban residence was associated with higher pregnancy rates (67%), though differences in residence were not statistically significant.

### 3.2 Behavioral and Sociocultural Factors‐Related Characteristics of Study Participants

In this study, as illustrated in Table 2, peer pressure showed a modest impact, with 40.1% of pregnant teens reporting it compared to 59.9% of nonpregnant teens. Contraceptive use is crucial, as only 13.2% of pregnant teens used contraception versus 86.8% of nonpregnant ones. Early marriage (68% vs. 25.8%) and having multiple sexual partners (73.3% vs. 26.3%) are strong risk factors. Educational challenges, parental strictness, and cultural attitudes also influence pregnancy rates, highlighting the need for targeted interventions.

### 3.3 Factors Associated With Teenage Pregnancy Among Girls

All the independent variables fit into the multivariate logistic regression model (Table 3) to identify factors associated with teenage pregnancy; only significant ones were recorded and tabulated. A priori factors associated with teenage pregnancy among girls included demographic variables such as age; adolescents aged 15–17 (AOR: 0.17, CI: 0.06–0.46) and 18–19 (AOR: 0.04, CI: 0.01–0.12) exhibited substantially lower odds of experiencing pregnancy compared to those aged 13–14. This suggests that older teenagers may have better access to education and resources. Educational attainment also played a vital role. Girls with no formal education have significantly lower odds of experiencing pregnancy (AOR: 0.16, CI: 0.03–0.80), emphasizing the protective effect of educational access. In contrast, those with secondary education show increased odds of pregnancy (AOR: 1.42, CI: 0.63–3.20), although this association is not statistically significant. Parental education level significantly influenced outcomes as well. Notably, adolescents whose parents have completed secondary education faced higher odds of teenage pregnancy (AOR: 3.50, CI: 1.50–8.15), indicating that parental guidance and support are critical in this context. Religious affiliation also emerged as a significant contributor. Muslim adolescents show notably higher odds of teenage pregnancy (AOR: 4.60, CI: 1.56–13.58), along with Protestants (AOR: 2.09, CI: 1.08–4.09), highlighting the influence of cultural factors on these outcomes. Furthermore, several societal factors were also strongly associated with increased odds of teenage pregnancy. Early marriage (AOR: 7.57, CI: 3.44–16.64), challenges with schooling (AOR: 6.97, CI: 3.18–15.31), and having strict parents (AOR: 6.37, CI: 2.54–15.98) all contributed significantly to the risk. Conversely, cultural acceptance of premarital sex is linked to lower odds of pregnancy (AOR: 0.16, CI: 0.038–0.64). These findings demonstrated the multifaceted nature of this public health issue (teenage pregnancy) and the need for comprehensive strategies to address it (Table 3).

## 4 Discussion

This study provided critical insights into the sociodemographic, behavioral, and cultural factors influencing teenage pregnancy among girls aged 13–19 in Apac District, Uganda. Our findings indicate a concerning prevalence of teenage pregnancy, particularly among younger adolescents, with 84.1% of pregnant participants aged 13–14. This finding underscores the importance of age as a significant determinant of teenage pregnancy. Previous research consistently highlights that younger adolescents face a higher risk of unintended pregnancies due to limited access to sexual and reproductive health education, information, and services [18]. These findings suggest that younger girls may experience substantial barriers to accessing the necessary reproductive health knowledge and services, thereby increasing their vulnerability to teenage pregnancy. This aligns with existing literature emphasizing the critical need for targeted education and service provision aimed at younger adolescents, particularly in rural settings.

Our study also found that educational attainment significantly influenced the likelihood of teenage pregnancy. The stark contrast between pregnancy rates of out‐of‐school girls (71.4%) and those with secondary education (26%) reinforces the protective role of education. This finding mirrors trends seen in other contexts, where education, especially secondary schooling, plays a pivotal role in reducing teenage pregnancy rates [19]. Educational access not only provides young women with sexual and reproductive health knowledge but also equips them with greater opportunities for economic independence, which further reduces their pregnancy risk. This result aligns with global evidence advocating for the expansion of education opportunities for girls, particularly in underserved areas. Thus, improving access to education, particularly in rural or impoverished areas, remains central to reducing teenage pregnancy rates.

The study corroborates previous research that highlights early marriage as a significant risk factor for teenage pregnancy. In this study, 68% of pregnant teens reported being married at an early age, which is consistent with trends seen in other sub‐Saharan African countries, including Tanzania [20]. Early marriage often leads to early pregnancies, driven by societal pressures and limited autonomy for girls. This highlights the importance of addressing early marriage through targeted interventions, such as legal frameworks, delayed marriage policies, and initiatives to challenge entrenched cultural norms. These efforts are crucial in reducing teenage pregnancy rates and enhancing the overall well‐being of young girls.

The low contraceptive use (13.2%) observed among pregnant teens in this study is consistent with findings from other sub‐Saharan African countries, where inadequate access to contraception contributes to higher rates of teenage pregnancy [21]. The lack of contraceptive use highlights significant barriers to accessing family planning services and information. These barriers include financial constraints, cultural attitudes, and misinformation about contraceptive methods. Given these challenges, the study emphasizes the urgent need for policy initiatives aimed at improving access to contraceptives and promoting comprehensive sexual and reproductive health education, both in schools and communities.

Interestingly, while studies have documented that higher parental education can increase the risk of teenage pregnancy due to diminished parental supervision and societal expectations [22], our study found that adolescents whose parents had secondary or postsecondary education had higher odds of teenage pregnancy. This finding suggests that parental education may be associated with complex, context‐specific dynamics that could affect adolescent pregnancy outcomes. Changes in parenting styles, socioeconomic status, and societal pressures on educated parents may inadvertently increase the risk of teenage pregnancy in certain contexts. This nuanced finding warrants further investigation to better understand the role of parental education and socioeconomic factors in shaping adolescent sexual behavior and pregnancy rates.

A striking finding from this study was that cultural acceptance of premarital sex was associated with lower odds of pregnancy. This finding contrasts with the common assumption that conservative cultural beliefs universally increase the risk of teenage pregnancy [23]. Instead, it suggests that cultural norms and societal attitudes toward premarital sex may have a more complex and varied influence on adolescent behavior. In regions where premarital sex is culturally accepted, interventions should focus on encouraging safe sexual practices, including consistent contraceptive use, rather than solely trying to change cultural norms. Future research should explore how cultural attitudes toward sex, contraception, and pregnancy shape adolescent sexual behavior and outcomes in different regions.

The findings of this study carry important implications for both policy and practice. First, there is an urgent need for comprehensive sexual and reproductive health education programs targeting both in‐school and out‐of‐school adolescents. These programs should provide accurate, age‐appropriate information on contraception, sexual rights, and the consequences of early pregnancy. Second, policies aimed at increasing educational access for girls, especially in rural and underserved areas, are crucial for reducing teenage pregnancies. Such policies should focus on reducing barriers to education, including socioeconomic constraints, gender norms, and cultural attitudes that discourage girls' education. Finally, community‐based programs addressing early marriage, cultural attitudes toward premarital sex, and contraceptive use should be prioritized. These interventions can play a pivotal role in reducing adolescent pregnancy rates and promoting the well‐being of young girls.

This study also contributes to the theoretical understanding of adolescent health by emphasizing the interconnectedness of sociodemographic, behavioral, and cultural factors in shaping teenage pregnancy. This holistic perspective can inform future research and interventions, encouraging interdisciplinary approaches to addressing teenage pregnancy as a public health challenge.

Future research should focus on exploring the underlying reasons for discrepancies in pregnancy rates among girls with postsecondary educated parents. It is important to examine the influence of peer groups, community norms, and social networks on adolescent sexual behavior, as these factors may provide additional insights into preventing teenage pregnancy. Furthermore, longitudinal studies are needed to assess the impact of educational interventions, policy changes, and community‐based programs over time. Such studies will help establish causal relationships and refine existing approaches to tackling adolescent pregnancy.

The strengths of this study include a robust sample size, which enhances the reliability and validity of the findings. The use of a descriptive cross‐sectional design allowed for comprehensive data collection on a variety of sociodemographic, behavioral, and cultural factors influencing teenage pregnancy. This broad approach facilitated a nuanced understanding of the complex issue of teenage pregnancy, and the large sample size provided sufficient statistical power for meaningful conclusions.

Despite these strengths, the study has several limitations. One key limitation is the potential for self‐reporting biases, especially regarding sensitive issues such as sexual behavior and contraceptive use. Adolescents may underreport or overreport their experiences due to social desirability or fear of judgment, which could affect the accuracy of the data. Additionally, the cross‐sectional nature of the study limits its ability to establish causal relationships between the identified factors and teenage pregnancy. Longitudinal studies would be beneficial in assessing the long‐term impact of educational interventions, policy changes, and community‐based programs on teenage pregnancy rates. Furthermore, the reliance on locally determined village populations may introduce sampling biases, as certain communities may be more likely to participate in research than others, potentially limiting the generalizability of the findings. Future studies with more diverse populations and longitudinal designs are necessary to confirm and expand upon the findings of this study.

## 5 Conclusion

This study demonstrates the multifaceted nature of teenage pregnancy in Apac District, Uganda, highlighting the critical roles of age, education, early marriage, and cultural influences in shaping pregnancy outcomes. The findings provide significant implications for policy and practice, emphasizing the need for targeted interventions that address educational access, delay early marriage, and promote contraceptive use among adolescents. Interventions that address these interconnected factors are crucial for improving maternal health outcomes and empowering young women in the region. The study suggests comprehensive measures to address teenage pregnancy in Apac District, Uganda. Recommendations include implementing comprehensive sexuality education, improving contraceptive access, engaging religious and community leaders, preventing early marriage, creating supportive school environments, and capacity building for healthcare providers. Additionally, ongoing research and monitoring efforts are crucial for evaluating interventions. By implementing these strategies collaboratively, stakeholders can effectively reduce teenage pregnancy rates and enhance the overall health and well‐being of adolescent girls in Apac District and similar contexts.

## Author Contributions

**Ewach Gracious Faith:** conceptualization, methodology, supervision, visualization, writing – original draft, writing – review and editing, data curation. **Emmanuel Asher Ikwara:** conceptualization, methodology, data curation, writing – review and editing, writing – original draft, software, formal analysis, visualization, supervision. **Musinguzi Marvin:** writing – original draft, writing – review and editing. **Isaac Isiko:** writing – original draft, writing – review and editing, methodology, data curation.

## Ethics Statement

The research conducted with human participants underwent a thorough review and received approval from the Gulu University Research Ethics Committee (REC), under protocol number GUREC‐2022‐198. Additionally, administrative clearance was granted by Lira University. It is worth noting that the REC responsible for approval was from Gulu University, as Lira University was in the process of establishing its own REC. During this period, studies conducted at Lira University were subjected to ethical review and clearance by the Gulu University REC, given their close affiliation.

## Consent

All participants involved in the study provided written informed consent before taking part.

## Conflicts of Interest

The authors declare no conflicts of interest.

## Transparency Statement

The lead author Ewach Gracious Faith affirms that this manuscript is an honest, accurate, and transparent account of the study being reported, that no important aspects of the study have been omitted, and that any discrepancies from the study as planned (and, if relevant, registered) have been explained.

## Data Availability

The data that support the findings of this study are available on request from the corresponding author. The data are not publicly available due to privacy or ethical restrictions.
